# Trends in Incidence of Conjunctival Melanoma in the US

**DOI:** 10.1001/jamanetworkopen.2022.37229

**Published:** 2022-10-18

**Authors:** Thomas A. Weppelmann, Keith T. Zimmerman, Vania Rashidi

**Affiliations:** 1 Department of Ophthalmology, Morsani College of Medicine, University of South Florida, Tampa; 2 James A. Haley Veterans’ Hospital Eye Clinic, Department of Veterans Affairs, Tampa, Florida; 3 Department of Ophthalmology and Visual Neurosciences, University of Minnesota, Minneapolis

## Abstract

This cohort study assesses the incidence of conjunctival melanoma, associations between demographic factors, and trends over time in the US.

## Introduction

Conjunctival melanoma (CM) is a rare ocular malignant neoplasm with a 5-year mortality rate of approximately 25% and local recurrence rate as high as 50%.^1^ Despite evidence that the incidence of CM increased during the later part of the 20th century, evaluations of these trends have not been published since 2003.^2^ In this cohort study, we assessed the current incidence of CM, associations between demographic factors, and trends over time in the US.

## Methods

Surveillance, Epidemiology, and End Results (SEER) data representative of 48% of the US population were compiled from January 1, 2000, to January 1, 2020, with 2 934 523 605 person-years of exposure.^3^ Incidence per 1 million people and 95% CIs were calculated as previously described.^4^ Incidence rate ratios (IRRs) compared each demographic category with the mean population incidence. Proxy income was estimated using the median household income from the county of residence. Trends in incidence by year of diagnosis were evaluated by linear regression expressed as annual percentage changes (APCs). Institutional review board approval was deemed unnecessary for use of publicly available, deidentified data from SEER. This study followed the STROBE reporting guideline. Data were analyzed from May 1 to July 1, 2022, using Stata software, version 12 (StataCorp LLC). Two-sided *P* < .05 indicated statistical significance.

## Results

**Figure 1.**
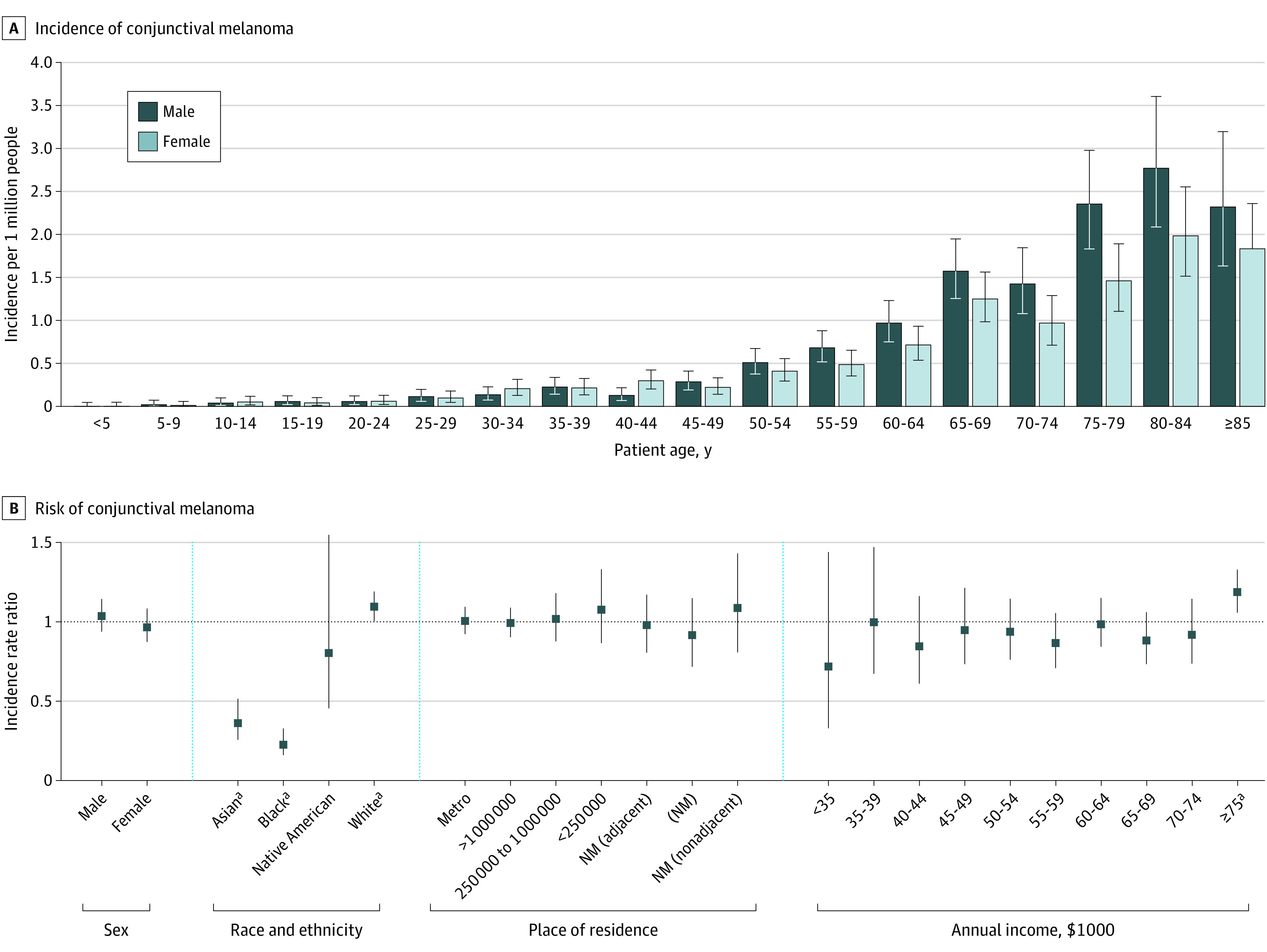
Incidence of Conjunctival Melanoma (CM) by Patient Age and Incidence Rate Ratios (IRRs) by Demographic Factors A, The incidence of CM per 1 million people is presented by age group with stratification by biological sex. B, The risk of CM expressed as IRRs are presented by demographic factors using age-adjusted incidence rates. The IRRs use the mean population incidence as the reference group. Error bars indicate 95% CIs. Metro indicates metropolitan; NM, nonmetropolitan. ^a^Significantly different than 1.0.

**Figure 2.**
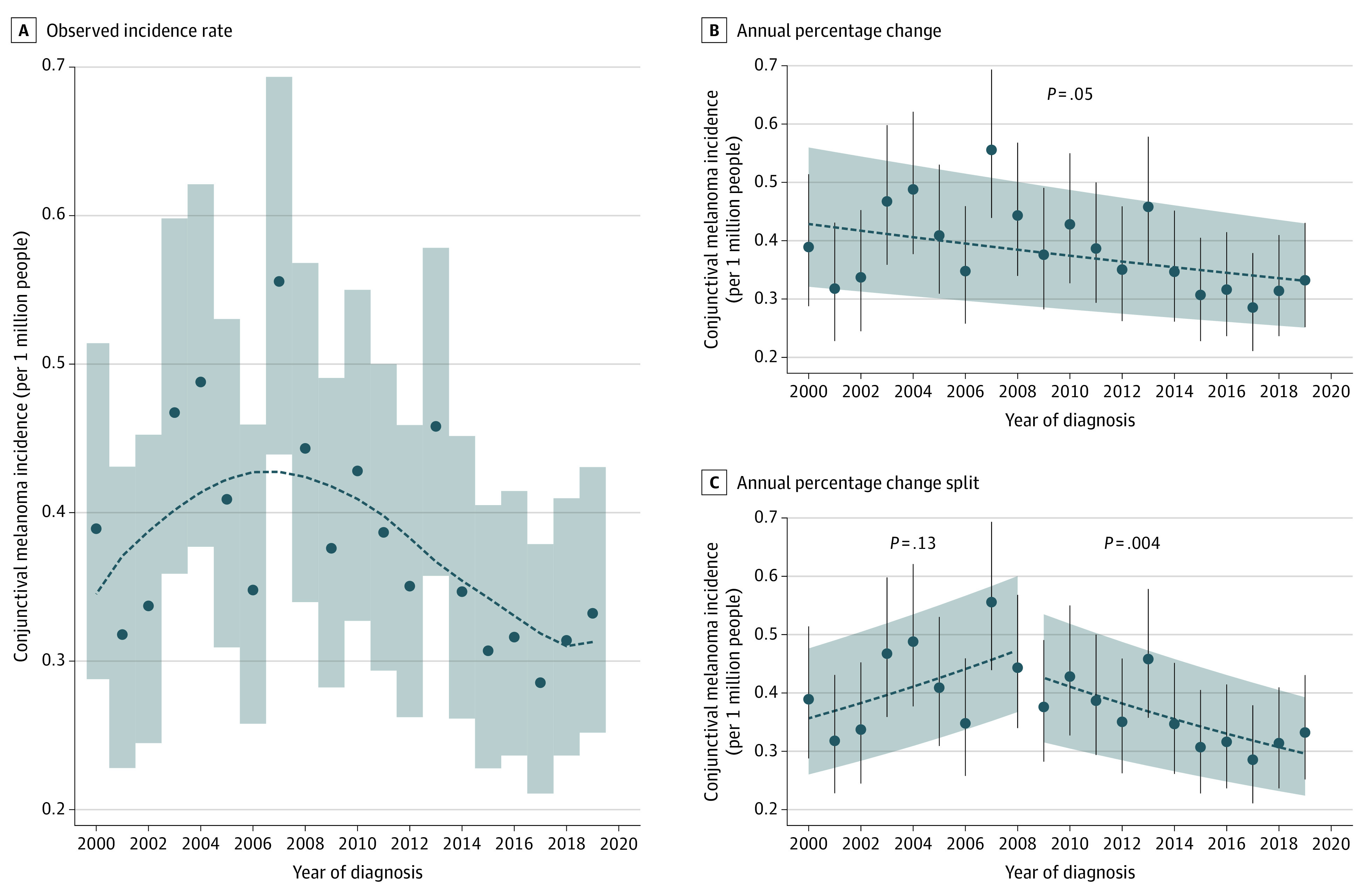
Trends in Conjunctival Melanoma (CM) Incidence by Year of Diagnosis The incidence of CM is presented by year of diagnosis with analyses of trends over time. A, Observed incidence rate per 1 million persons adjusted for age (dots), 95% CIs (gray shading), and locally estimated scatterplot smoothing line to visualize the pattern (blue dashed line). B and C, Linear regression models show the incidence by year with the observed incidence and age-adjusted 95% CIs (dots with whiskers), the estimated linear trend (dashed lines), the 95% CIs for the trend (gray shading), and significance testing (*P* values) for the null hypothesis that the slope is different than zero.

Overall, 1147 individuals were diagnosed with CM (585 men [51.0%] and 562 women [49.0%]; 903 [78.7%] were aged ≥50 years). Race data were collected by SEER on the case-reporting form because racial differences are significant for both cutaneous and conjunctival melanoma incidence. Fewer than 1% of individuals were American Indian or Alaskan Native; 31 (2.7%) were Asian or Pacific Islander, 28 (2.4%) were Black, and 1046 (91.2%) were White. The crude incidence and age-adjusted incidence rates were 0.39 (95% CI, 0.33-0.45) and 0.38 (95% CI, 0.36-0.40) per million people, respectively. Incidence of CM increased with age, was lowest among Black (IRR, 0.23 [95% CI, 0.16-0.33]) and Asian or Pacific Islander (IRR, 0.36 [95% CI, 0.26-0.52]) individuals, and was highest among White individuals (IRR, 1.11 [95% CI, 1.01-1.20]; *P* < .001 for all) (Figure 1). The incidence was lowest in those making less than $35 000 per year (but not significantly different), highest in those making more than $75 000 per year (IRR, 1.19 [95% CI, 1.06-1.33]; *P* < .001), and not significantly different by sex or place of residence. Between 2000 and 2020, CM incidence decreased from 0.39 to 0.33 per million (Figure 2). The APC decreased by 1.4% (95% CI, −2.7% to −0.01%; *P* = .05). Splitting the study period into 2 regression models revealed a nonsignificant APC increase of 3.5% (95% CI, −1.7% to 8.7%) from 2000 to 2008 (*P* = .15), followed by a significant decrease of 3.3% (95% CI, −1.3% to −5.2%) from 2009 to 2020 (*P* = .004).

## Discussion

In contrast to CM incidence between 1973 and 1999, which increased 5.5% every 2 years,^2^ this cohort study found CM incidence between 2000 and 2008 stabilized and then decreased by 3.3% per year until 2020. We speculate that increased physician awareness of CM during the late 20th century led to a more accurate estimation of the true disease burden, which was previously underdiagnosed. Although increased physician awareness is likely considering the rising number of relevant publications since the 1970s, a limitation of this study is the lack of data on the number of biopsies performed to correlate changes in awareness with clinical practice. As with cutaneous melanoma, the subsequent decline in incidence may be attributable to increased public awareness regarding skin cancer and reduced UV light exposure.^5^ Additionally, although associations among CM incidence, age, and race and ethnicity endure,^6^ this study identified differences in CM incidence by income level. This observation could be due to differences in patient risk factors by income levels or indicate a health disparity in the detection of CM for low-income patients with reduced health care access. As with other cancers, such disparities may contribute to more advanced disease at diagnosis and reduced survival and therefore warrant further investigation.
